# Supplementary material for: Gene flow contributes to diversification of the major fungal pathogen Candida albicans
Source: Nat Commun. 2018 Jun 8;9:2253. doi: 10.1038/s41467-018-04787-4 (PMC5993739; doi:10.1038/s41467-018-04787-4)
Supplement: Supplementary file 1 — Supplementary Information [file 41467_2018_4787_MOESM1_ESM.pdf]

Gene flow contributes to diversification of the major fungal pathogen *Candida albicans*.

Ropars et al.

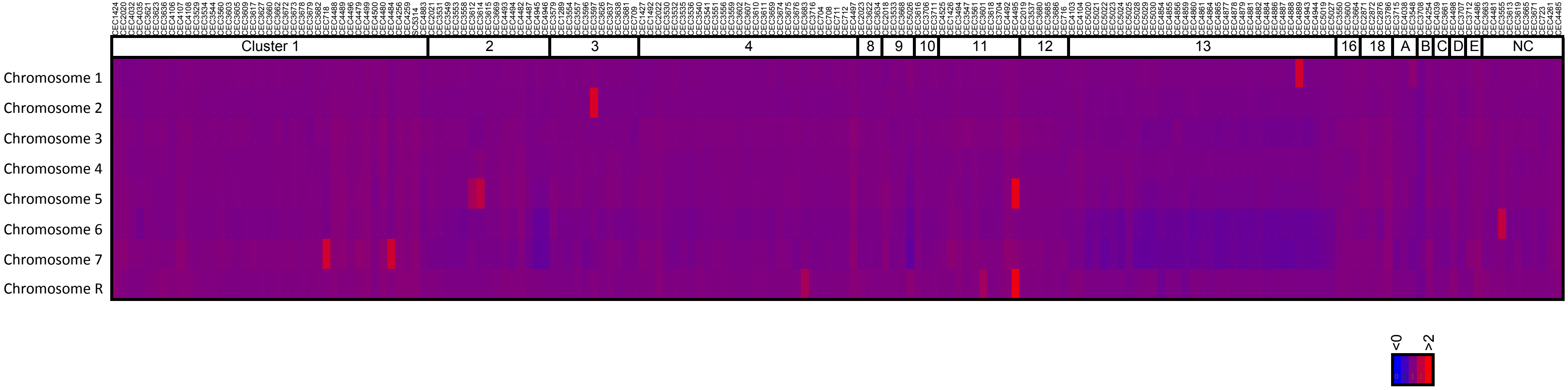

Supplementary Fig. 1: Heatmap representing average sequencing depth on the eight chromosomes for the 182 isolates of *C.albicans.*

Sequencing depth obtained for each bin of 1kb on each chromosome was multiplied by the ploidy of the strain as defined from FACS analysis, divided by the genome sequencing depth and converted to log2 values. These values were then corrected through division by the median of all values obtained for chromosomes that had an average sequencing depth that did not deviate by more than 20% from the average sequencing depth of the whole genome. Average of the values obtained for each chromosome is shown, with diploid chromosome at ~1, triploid chromosomes at ~1.58 and tetraploid chromosomes at ~2. The color scale is indicated. While examples of triploid or tetraploid chromosomes are observed, no example of haploid chromosome could be seen. We removed the three strains that are not diploid (see Supplementary Table 1).

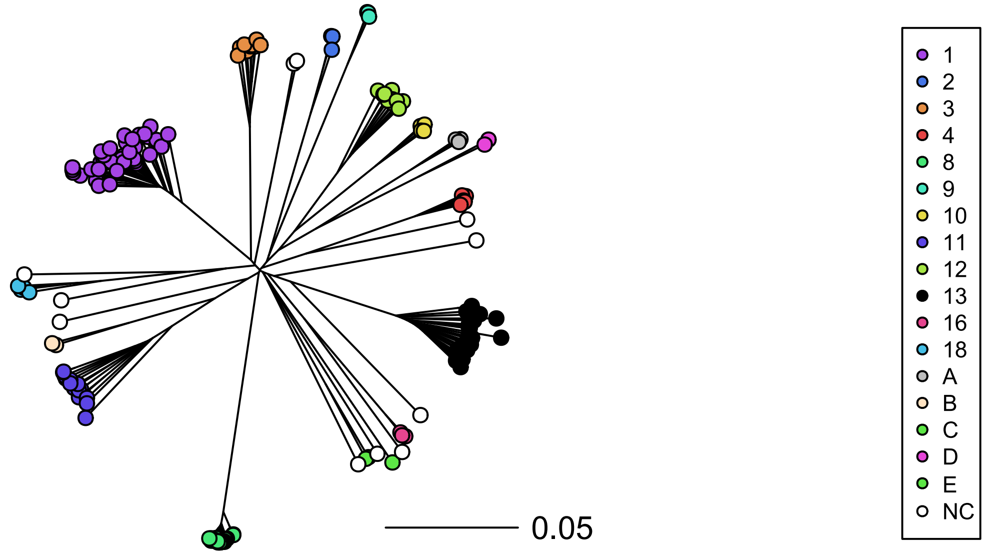

Supplementary Fig. 2 (related to Fig. 2): Distance tree based on 183,974 insertion-deletion events (indels) information.

Insertions at homozygous state were coded as 2, insertions at heterozygous state as 1 and no indel event as 0. Branch length are shown and the scale bar represents 0.05 number of differences per site.

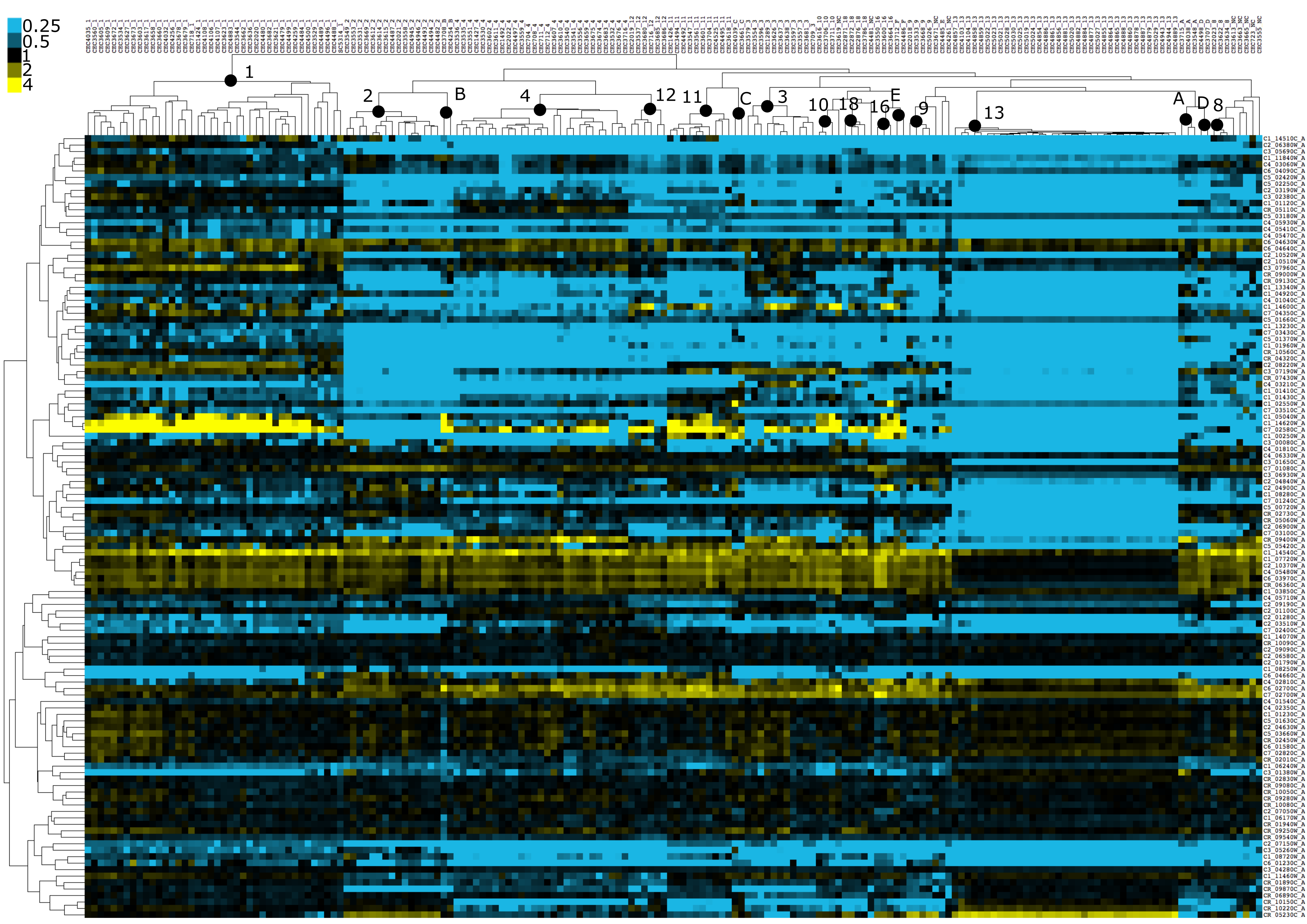
0.25
0.5
1
2
4
1
2
B
4
12
11
C
3
10
18
16
E
9
13
A
D
8

Supplementary Fig. 3 (related to Fig. 2): Hierarchical clustering of the 182 isolates of *C. albicans* based on the coverage of repeat regions (including long-terminal repeats, retrotransposons and repeat regions) normalized by chromosome.

The clustering was generated by Cluster 3.0 using hierarchical clustering (complete linkage clustering) and the spearman rank correlation for measuring non-parametric distance, and visualized with java treeview by converting values in log2 scale. The scale bar on the top left indicates color codes.

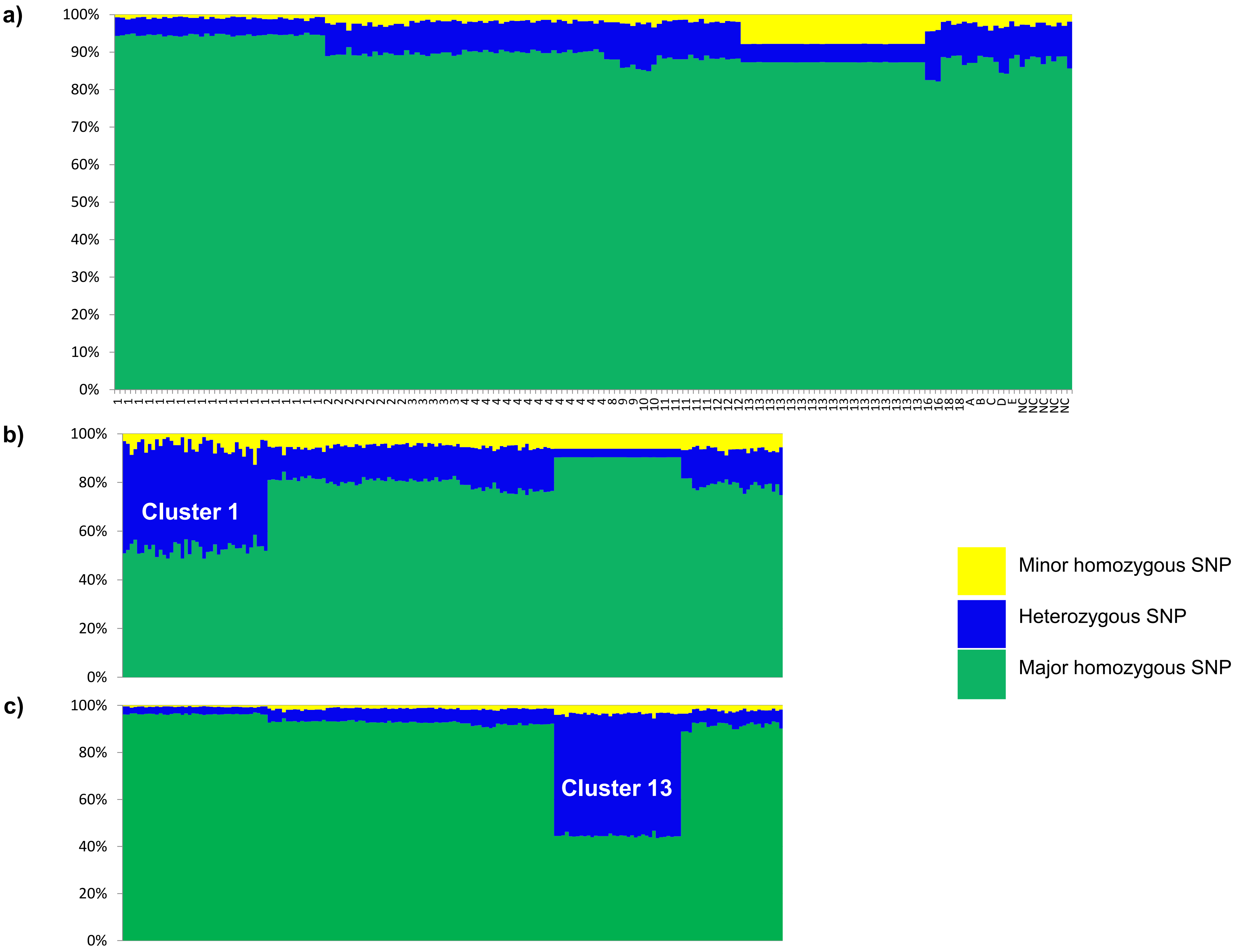
a)
100%
90%
80%
70%
60%
50%
40%
30%
20%
10%
0%
b)
100%
80%
60%
40%
20%
0%
Cluster 1
c)
100%
80%
60%
40%
20%
0%
Cluster 13
Minor homozygous SNP
Heterozygous SNP
Major homozygous SNP

Supplementary Fig. 4: Graphic representation of counts of major / minor homozygous SNPs and heterozygous SNPs at a) the whole population level (182 isolates, 262,661 SNPs) and b) and c) at the cluster level.

b) SNP positions invariant across cluster 1 were removed, the analysis thus included 28,142 polymorphic sites; c) SNP positions invariant across cluster 13 were removed, the analysis thus included 12,096 polymorphic sites. This shows an excess of homozygous SNPs at the whole population level and an excess of heterozygous SNPs within each cluster (shown here: clusters 1 and 13).

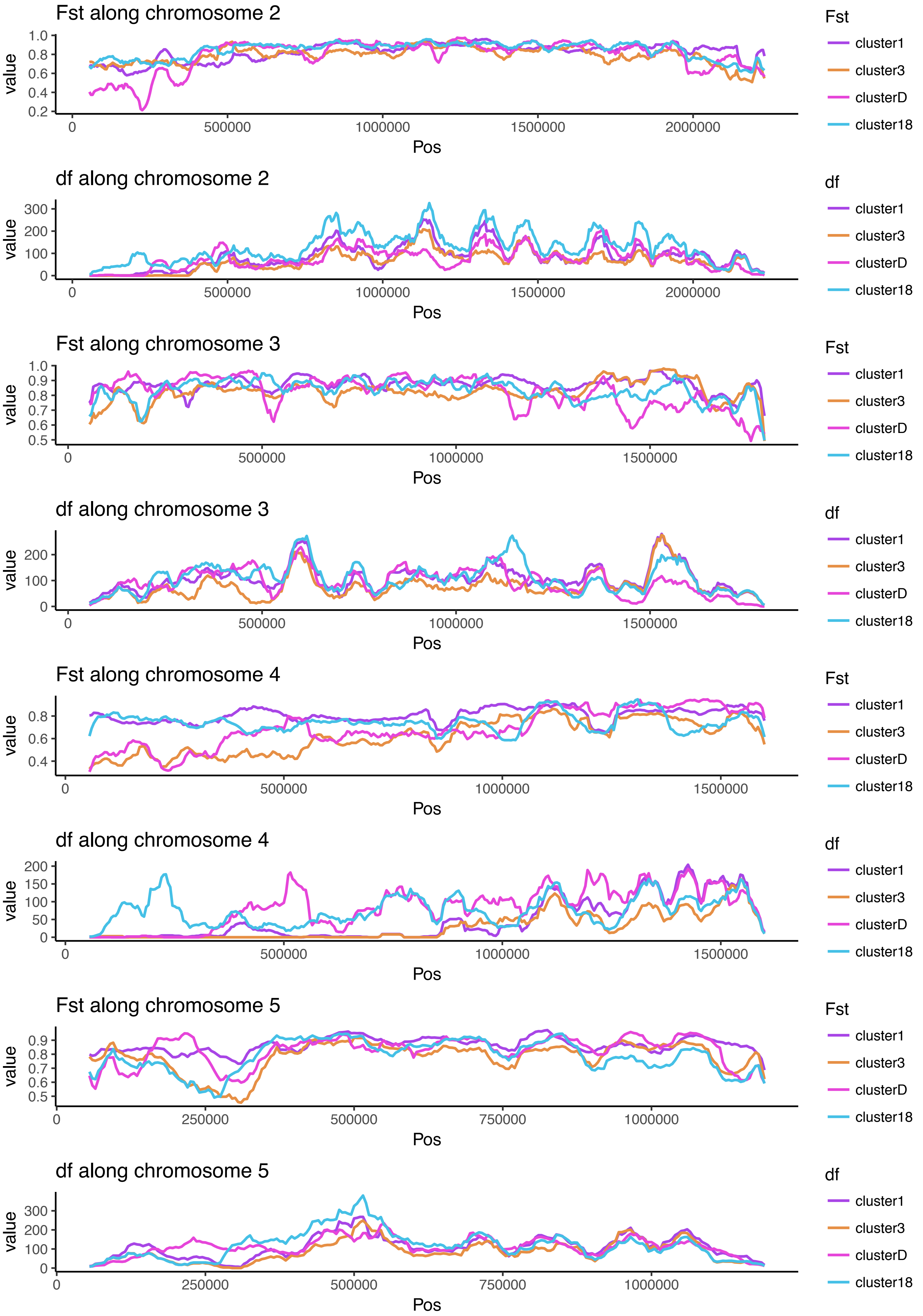
Fst along chromosome 2
value
1.0
0.8
0.6
0.4
0.2
0
500000
1000000
1500000
2000000
Pos
Fst
cluster1
cluster3
clusterD
cluster18
df along chromosome 2
value
300
200
100
0
0
500000
1000000
1500000
2000000
Pos
df
cluster1
cluster3
clusterD
cluster18
Fst along chromosome 3
value
1.0
0.9
0.8
0.7
0.6
0.5
0
500000
1000000
1500000
Pos
Fst
cluster1
cluster3
clusterD
cluster18
df along chromosome 3
value
200
100
0
0
500000
1000000
1500000
Pos
df
cluster1
cluster3
clusterD
cluster18
Fst along chromosome 4
value
0.8
0.6
0.4
0
500000
1000000
1500000
Pos
Fst
cluster1
cluster3
clusterD
cluster18
df along chromosome 4
value
200
150
100
50
0
0
500000
1000000
1500000
Pos
df
cluster1
cluster3
clusterD
cluster18
Fst along chromosome 5
value
0.9
0.8
0.7
0.6
0.5
0
250000
500000
750000
1000000
Pos
Fst
cluster1
cluster3
clusterD
cluster18
df along chromosome 5
value
300
200
100
0
0
250000
500000
750000
1000000
Pos
df
cluster1
cluster3
clusterD
cluster18

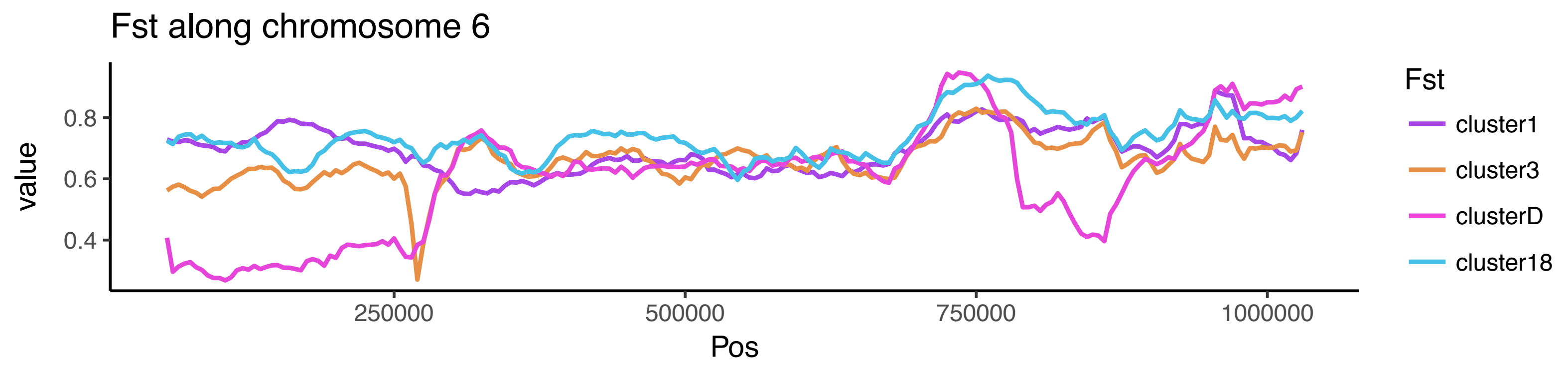
Fst along chromosome 6
value
0.8
0.6
0.4
250000
500000
750000
1000000
Pos
Fst
cluster1
cluster3
clusterD
cluster18

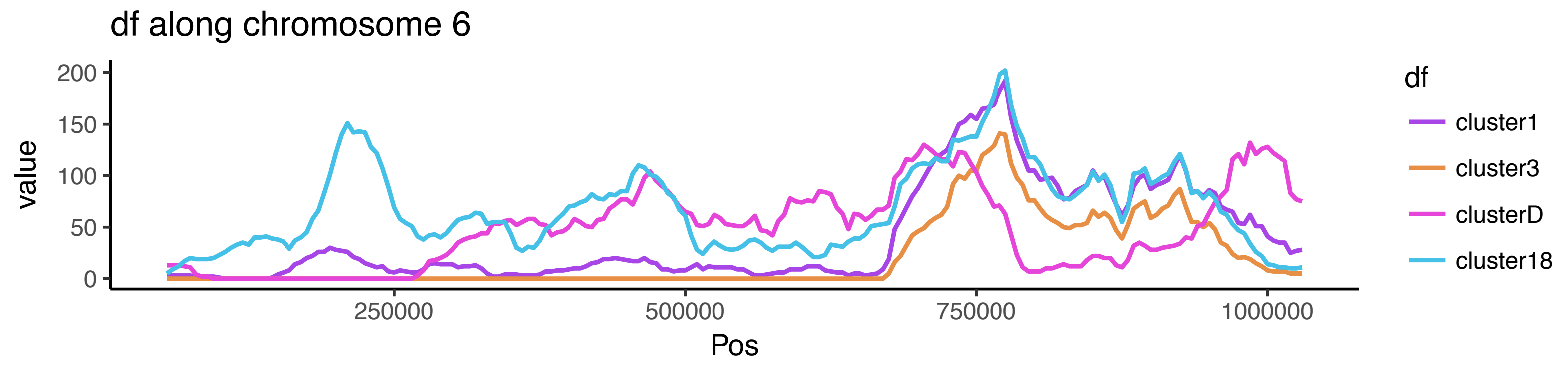
df along chromosome 6
value
200
150
100
50
0
250000
500000
750000
1000000
Pos
df
cluster1
cluster3
clusterD
cluster18

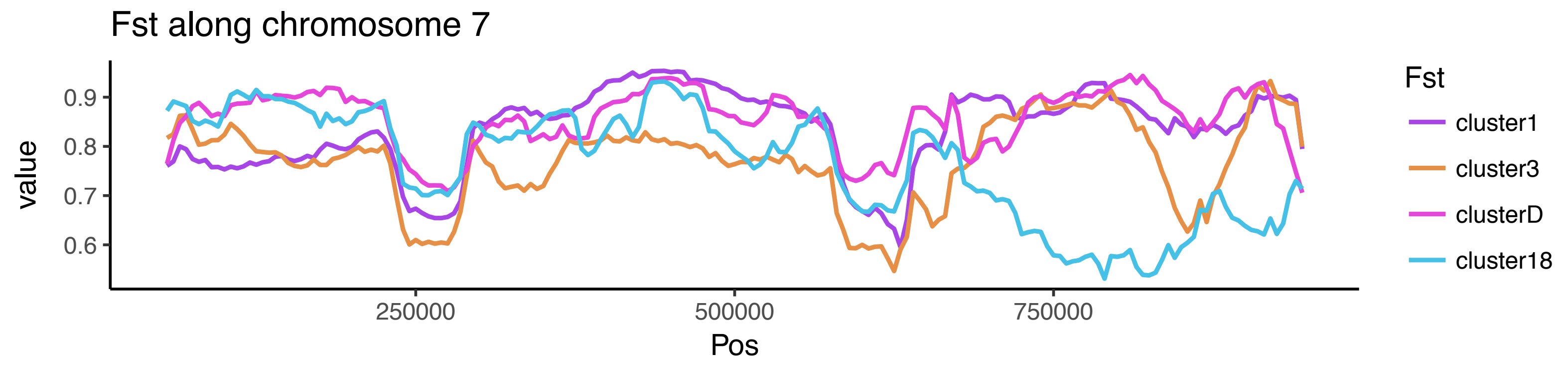
Fst along chromosome 7
value
0.9
0.8
0.7
0.6
250000
500000
750000
Pos
Fst
cluster1
cluster3
clusterD
cluster18

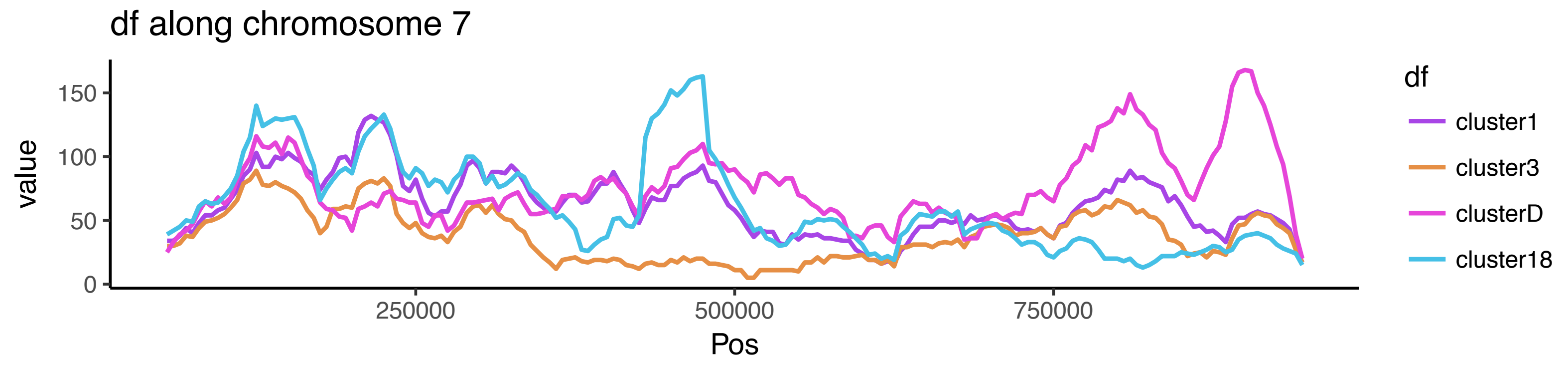
df along chromosome 7
value
150
100
50
0
250000
500000
750000
Pos
df
cluster1
cluster3
clusterD
cluster18

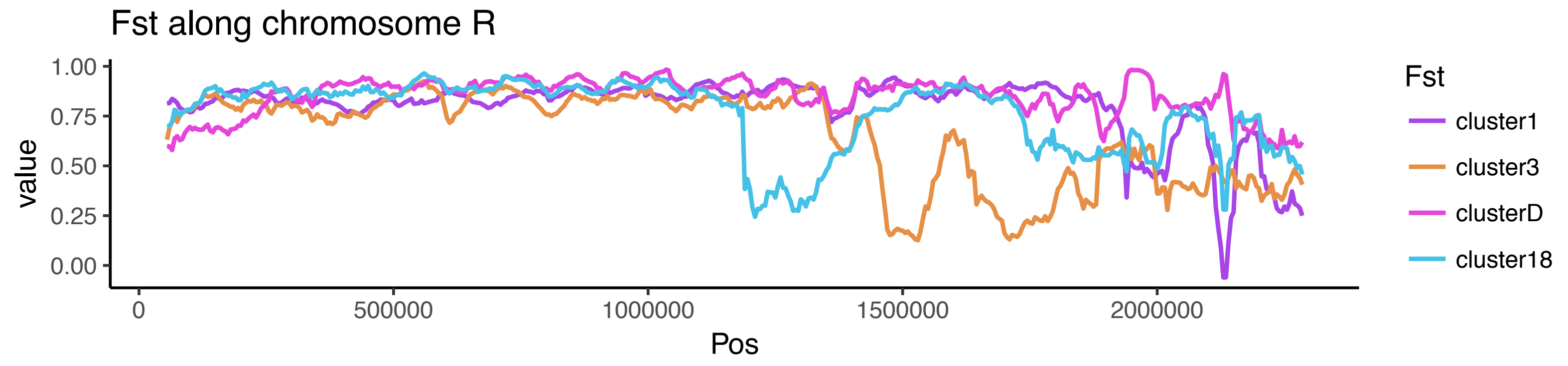
Fst along chromosome R
value
1.00
0.75
0.50
0.25
0.00
0
500000
1000000
1500000
2000000
Pos
Fst
cluster1
cluster3
clusterD
cluster18

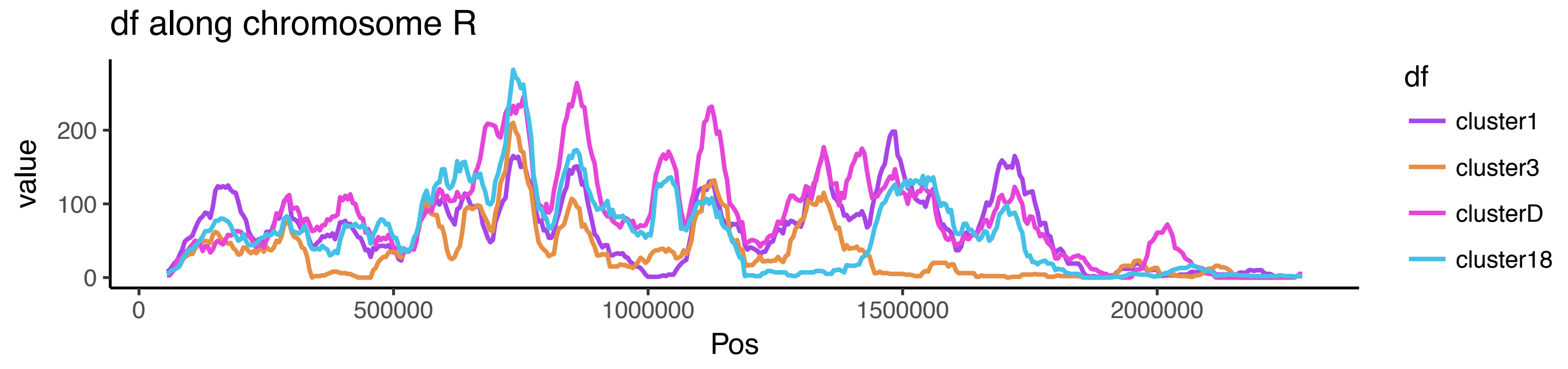
df along chromosome R
value
200
100
0
0
500000
1000000
1500000
2000000
Pos
df
cluster1
cluster3
clusterD
cluster18

Supplementary Fig. 5 (related to Fig. 4): Genomic scans of FST (fixation index, an index measuring the differentiation between populations) and df (the number of fixed differences between populations) in sliding windows of 50 kb using a window step of 5,000 bp of cluster A compared to clusters 1, 3, D and 18 along chromosomes 2 to 7 and R.

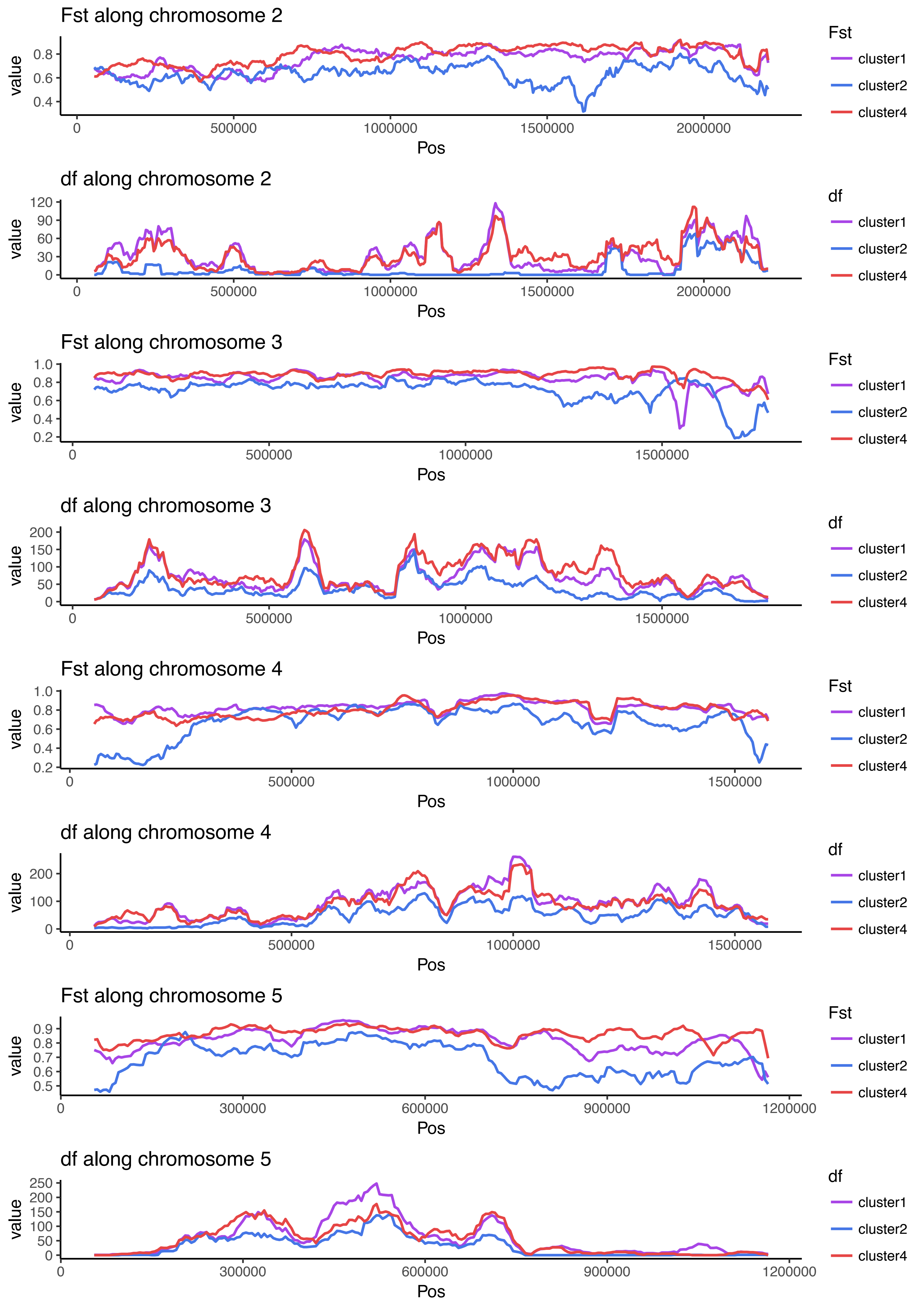
Fst along chromosome 2
value
0.4
0.6
0.8
0
500000
1000000
1500000
2000000
Pos
Fst
cluster1
cluster2
cluster4
df along chromosome 2
value
0
30
60
90
120
0
500000
1000000
1500000
2000000
Pos
df
cluster1
cluster2
cluster4
Fst along chromosome 3
value
0.2
0.4
0.6
0.8
1.0
0
500000
1000000
1500000
Pos
Fst
cluster1
cluster2
cluster4
df along chromosome 3
value
0
50
100
150
200
0
500000
1000000
1500000
Pos
df
cluster1
cluster2
cluster4
Fst along chromosome 4
value
0.2
0.4
0.6
0.8
1.0
0
500000
1000000
1500000
Pos
Fst
cluster1
cluster2
cluster4
df along chromosome 4
value
0
100
200
0
500000
1000000
1500000
Pos
df
cluster1
cluster2
cluster4
Fst along chromosome 5
value
0.5
0.6
0.7
0.8
0.9
0
300000
600000
900000
1200000
Pos
Fst
cluster1
cluster2
cluster4
df along chromosome 5
value
0
50
100
150
200
250
0
300000
600000
900000
1200000
Pos
df
cluster1
cluster2
cluster4

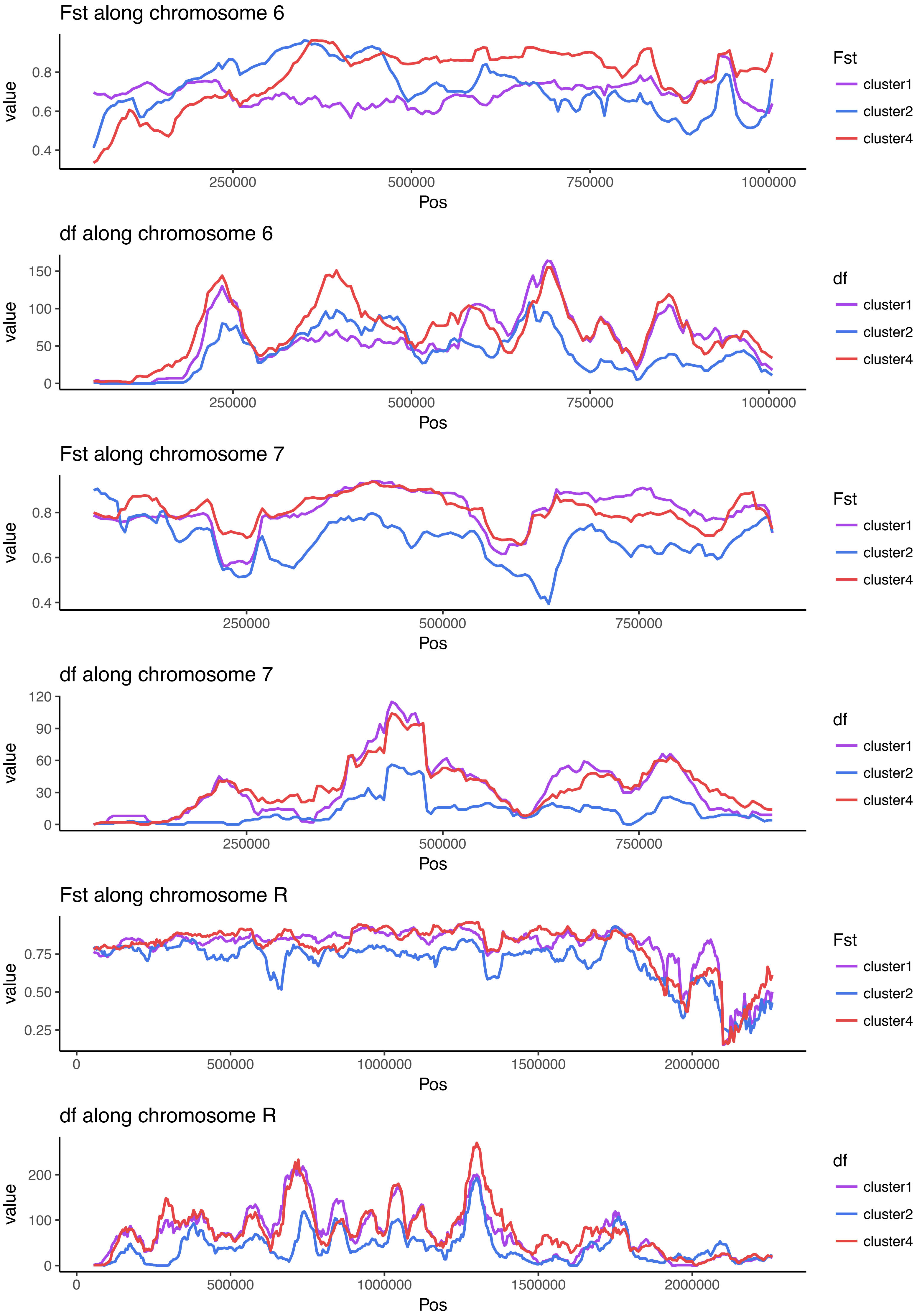

Fst along chromosome 6
value
0.4
0.6
0.8
250000
500000
750000
1000000
Pos
Fst
cluster1
cluster2
cluster4
df along chromosome 6
value
0
50
100
150
250000
500000
750000
1000000
Pos
df
cluster1
cluster2
cluster4
Fst along chromosome 7
value
0.4
0.6
0.8
250000
500000
750000
Pos
Fst
cluster1
cluster2
cluster4
df along chromosome 7
value
0
30
60
90
120
250000
500000
750000
Pos
df
cluster1
cluster2
cluster4
Fst along chromosome R
value
0.25
0.50
0.75
0
500000
1000000
1500000
2000000
Pos
Fst
cluster1
cluster2
cluster4
df along chromosome R
value
0
100
200
0
500000
1000000
1500000
2000000
Pos
df
cluster1
cluster2
cluster4

Supplementary Fig. 6 (related to Fig. 4): Genomic scans of FST (fixation index, an index measuring the differentiation between populations) and df (the number of fixed differences between populations) in sliding windows of 50 kb using a window step of 5,000 bp of cluster B compared to clusters 2 and B along chromosomes 2 to 7 and R.

CHROMOSOME 1: sliding windows of 50,000bp, step 5000

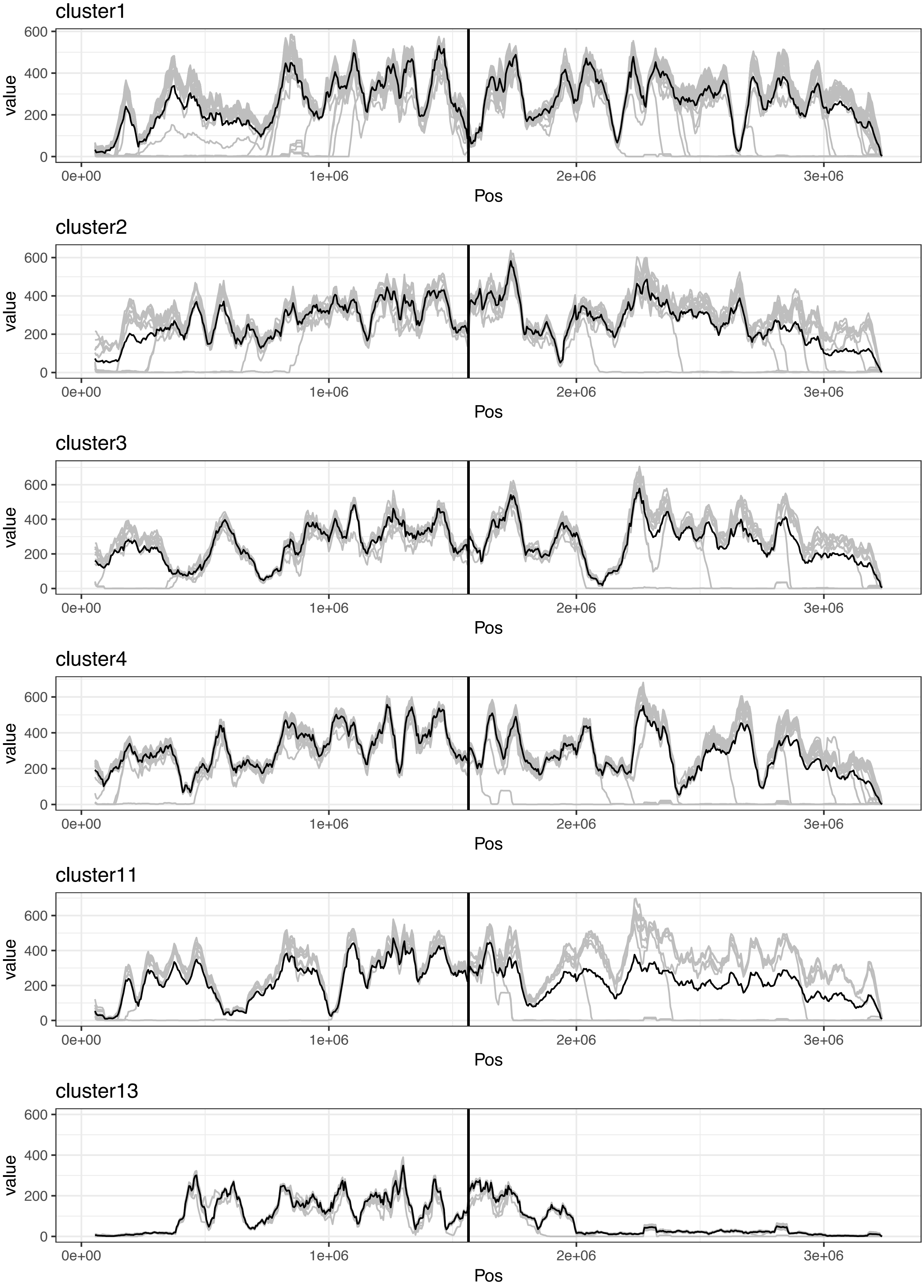

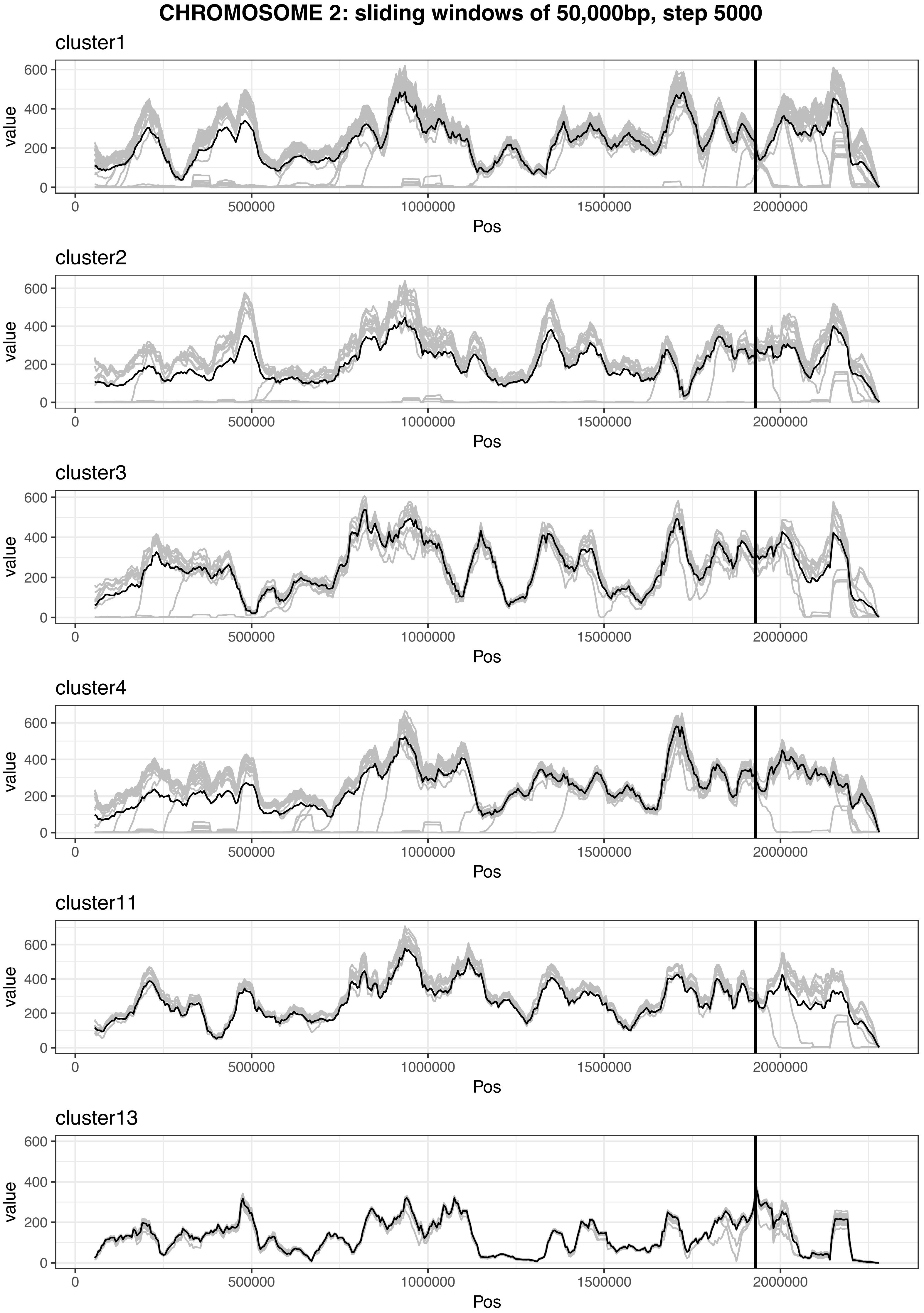
CHROMOSOME 2: sliding windows of 50,000bp, step 5000
cluster1
cluster2
cluster3
cluster4
cluster11
cluster13
value
Pos
0
200
400
600
0
500000
1000000
1500000
2000000

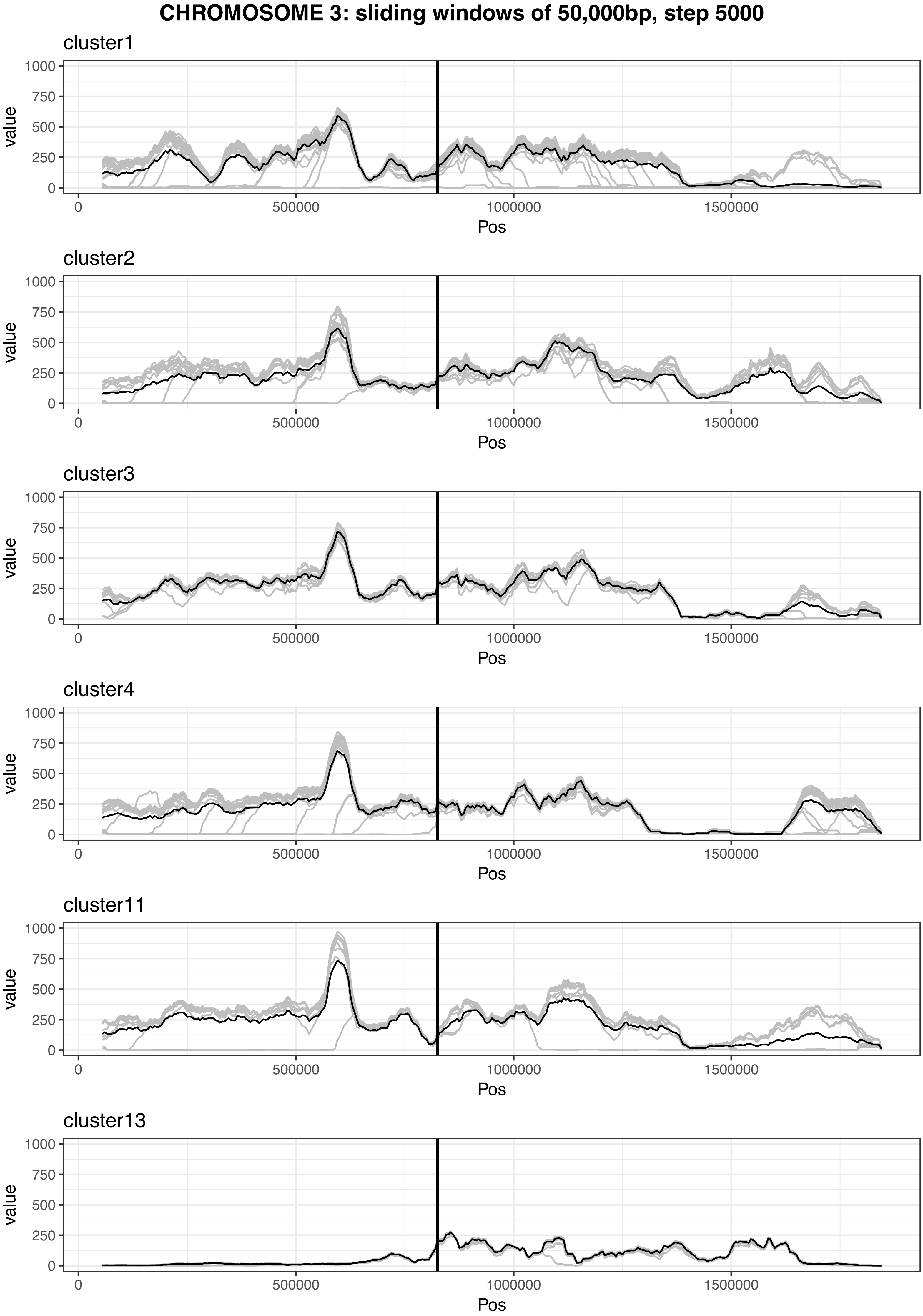
CHROMOSOME 3: sliding windows of 50,000bp, step 5000
cluster1
cluster2
cluster3
cluster4
cluster11
cluster13
value
0
250
500
750
1000
Pos
0
500000
1000000
1500000

CHROMOSOME 4: sliding windows of 50,000bp, step 5000

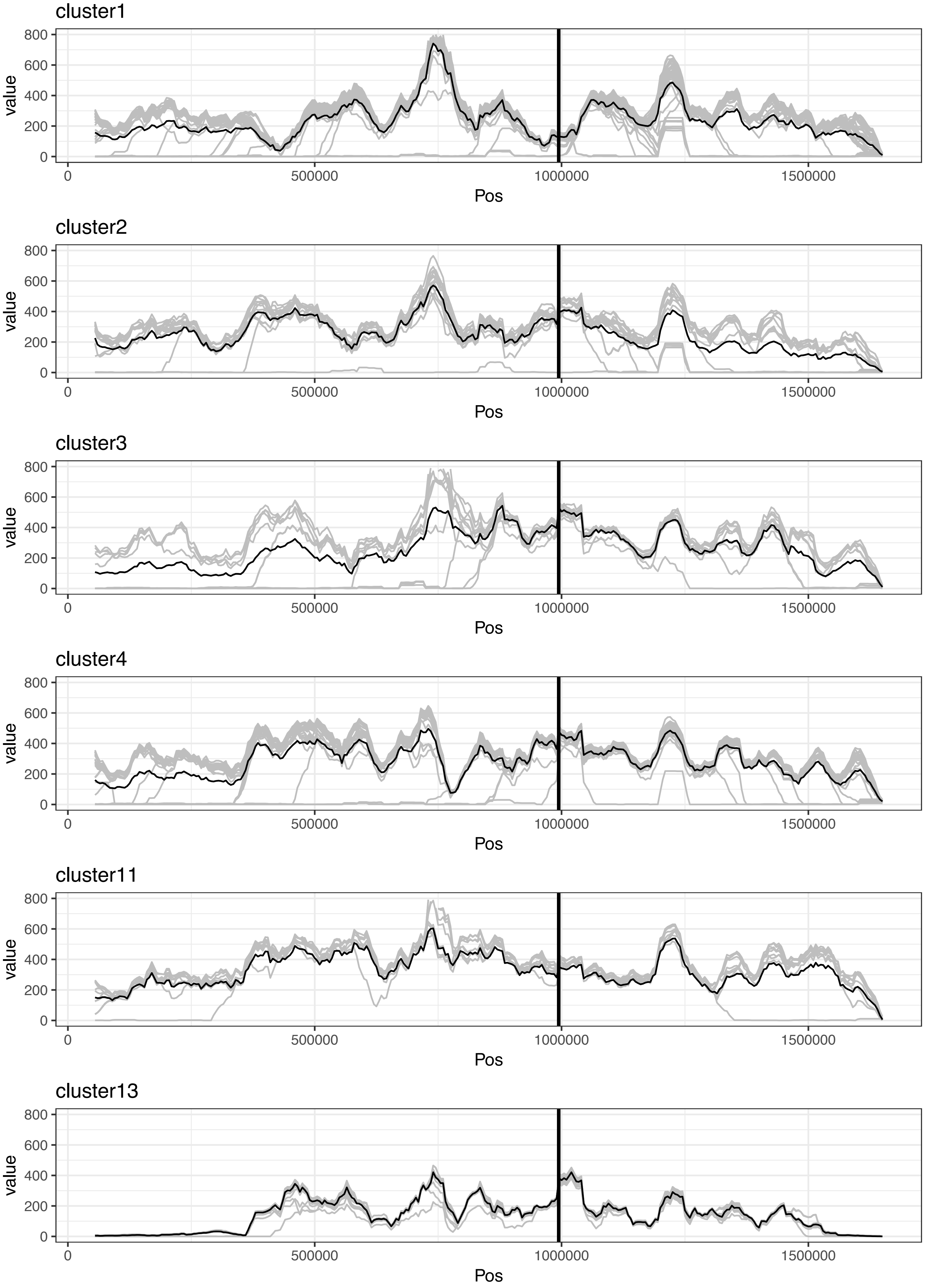

CHROMOSOME 5: sliding windows of 50,000bp, step 5000

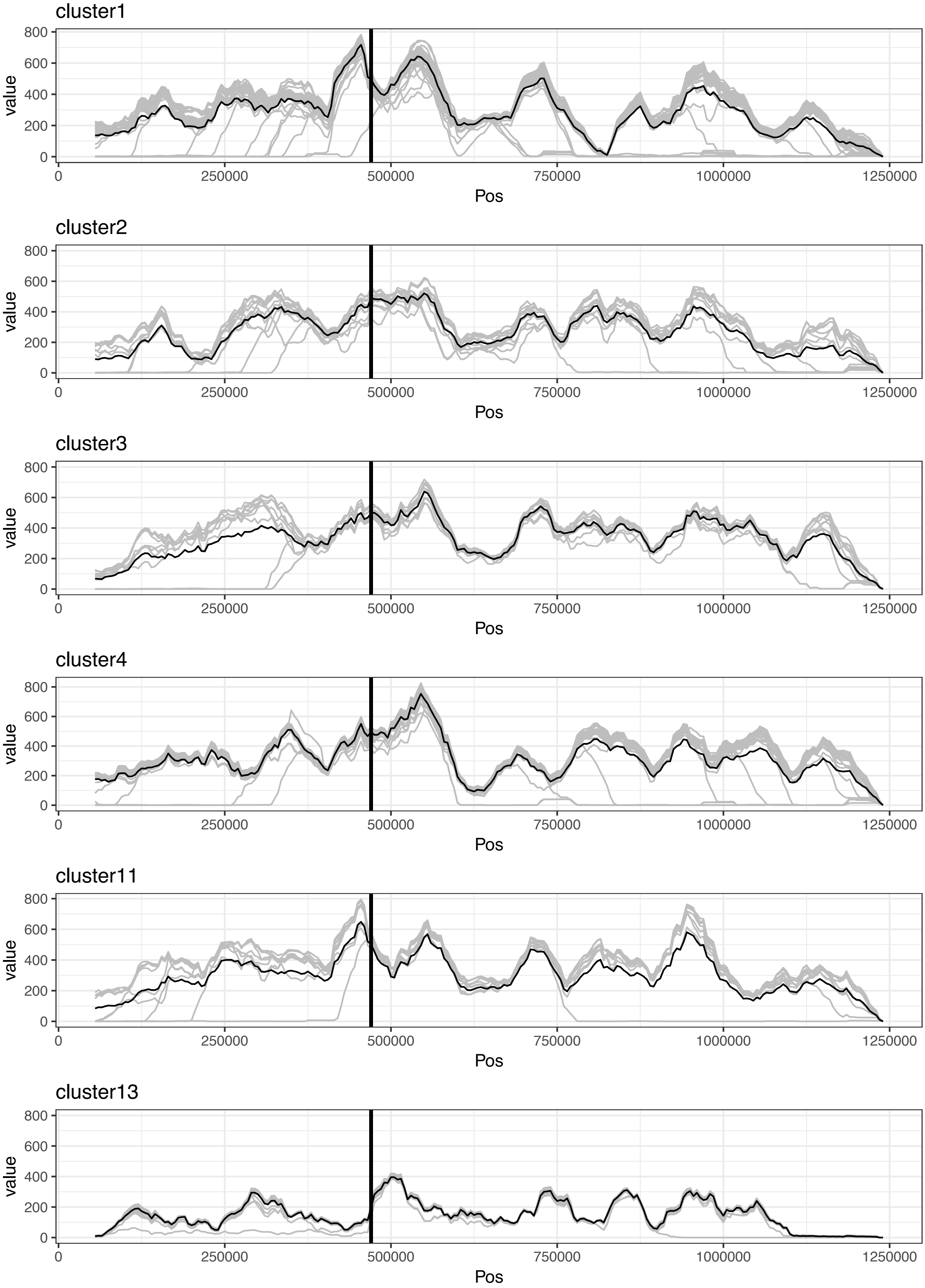

CHROMOSOME 6: sliding windows of 50,000bp, step 5000

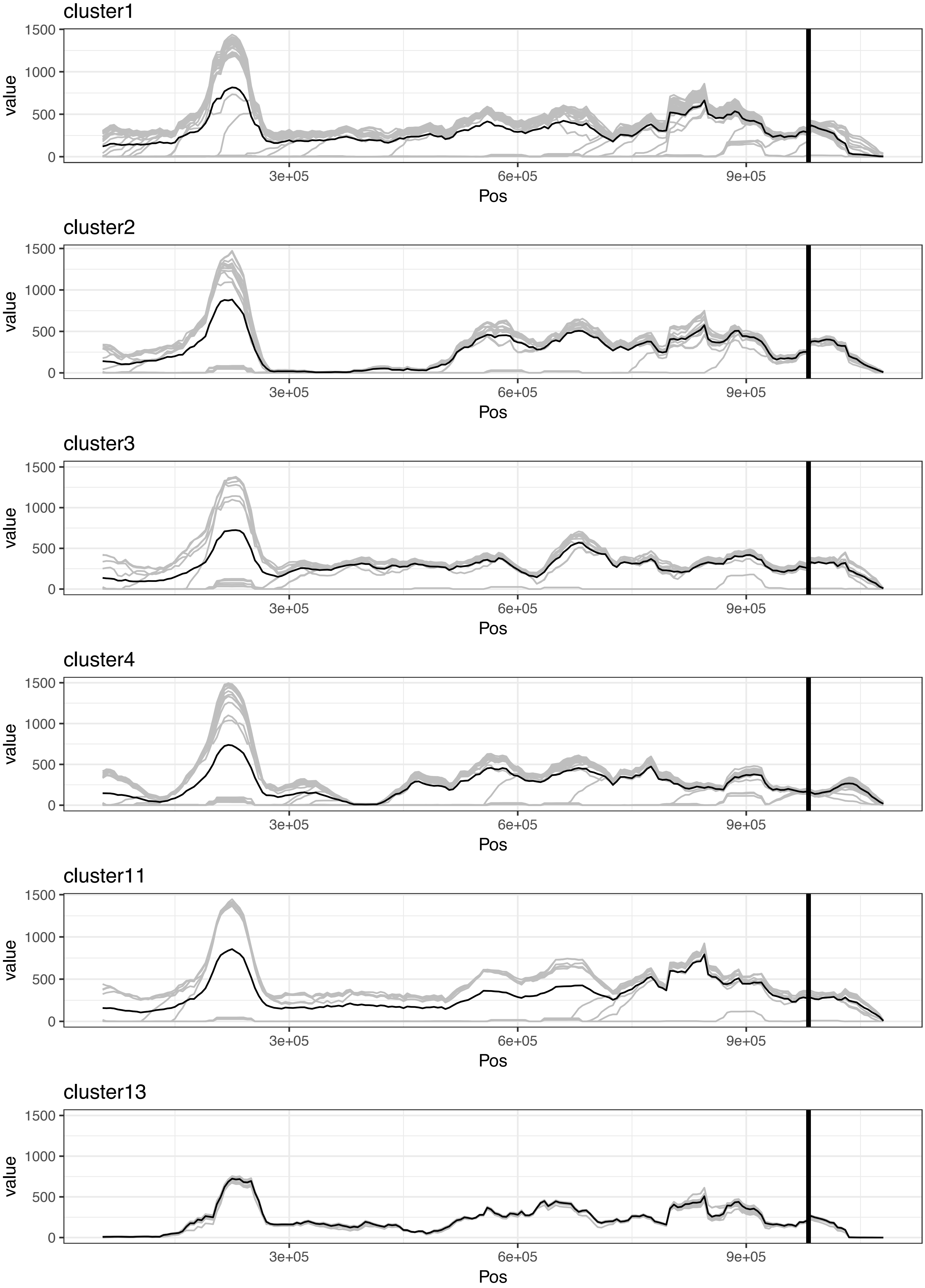

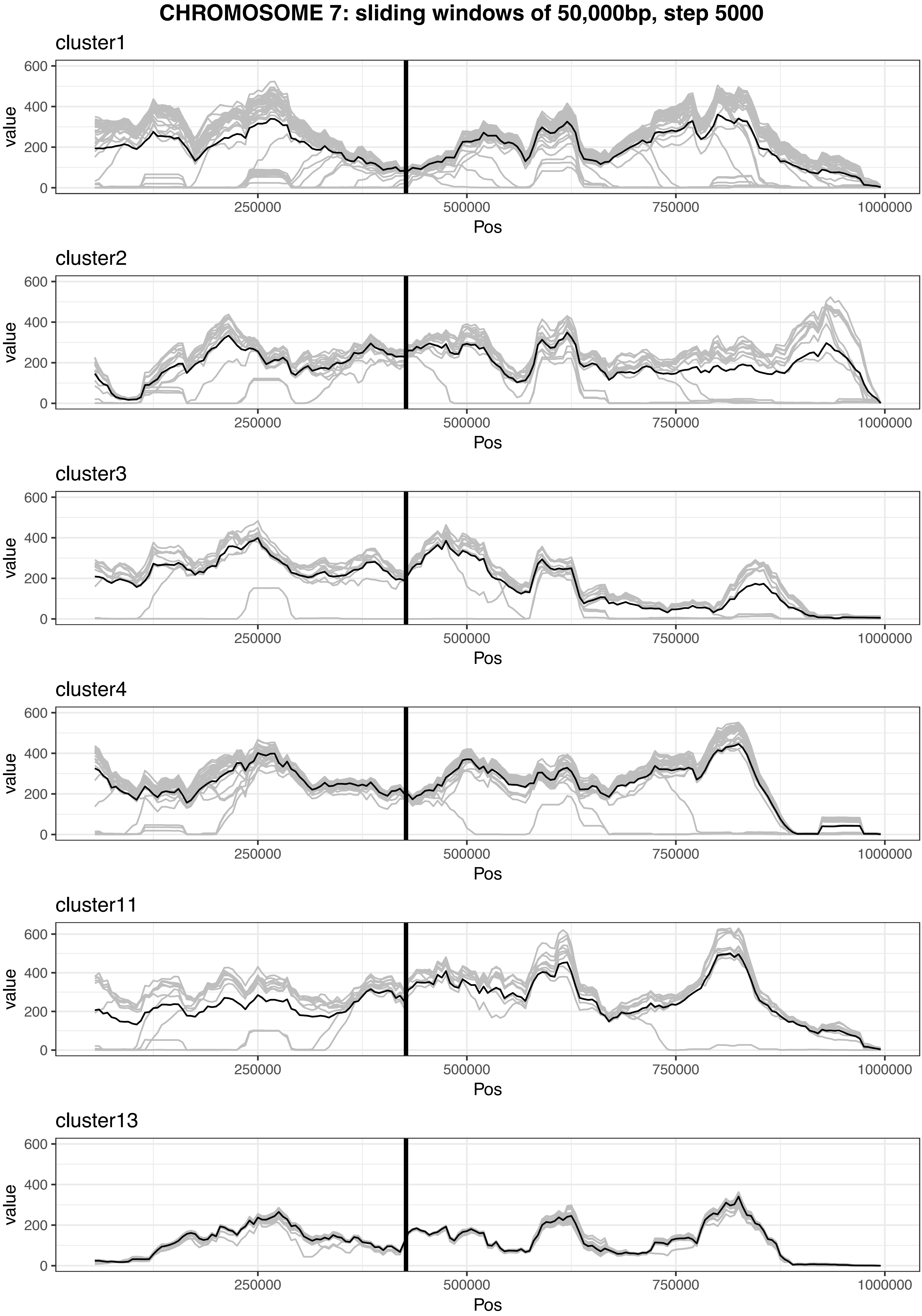
CHROMOSOME 7: sliding windows of 50,000bp, step 5000
cluster1
cluster2
cluster3
cluster4
cluster11
cluster13
value
Pos
0
200
400
600
250000
500000
750000
1000000

CHROMOSOME R: sliding windows of 50,000bp, step 5000

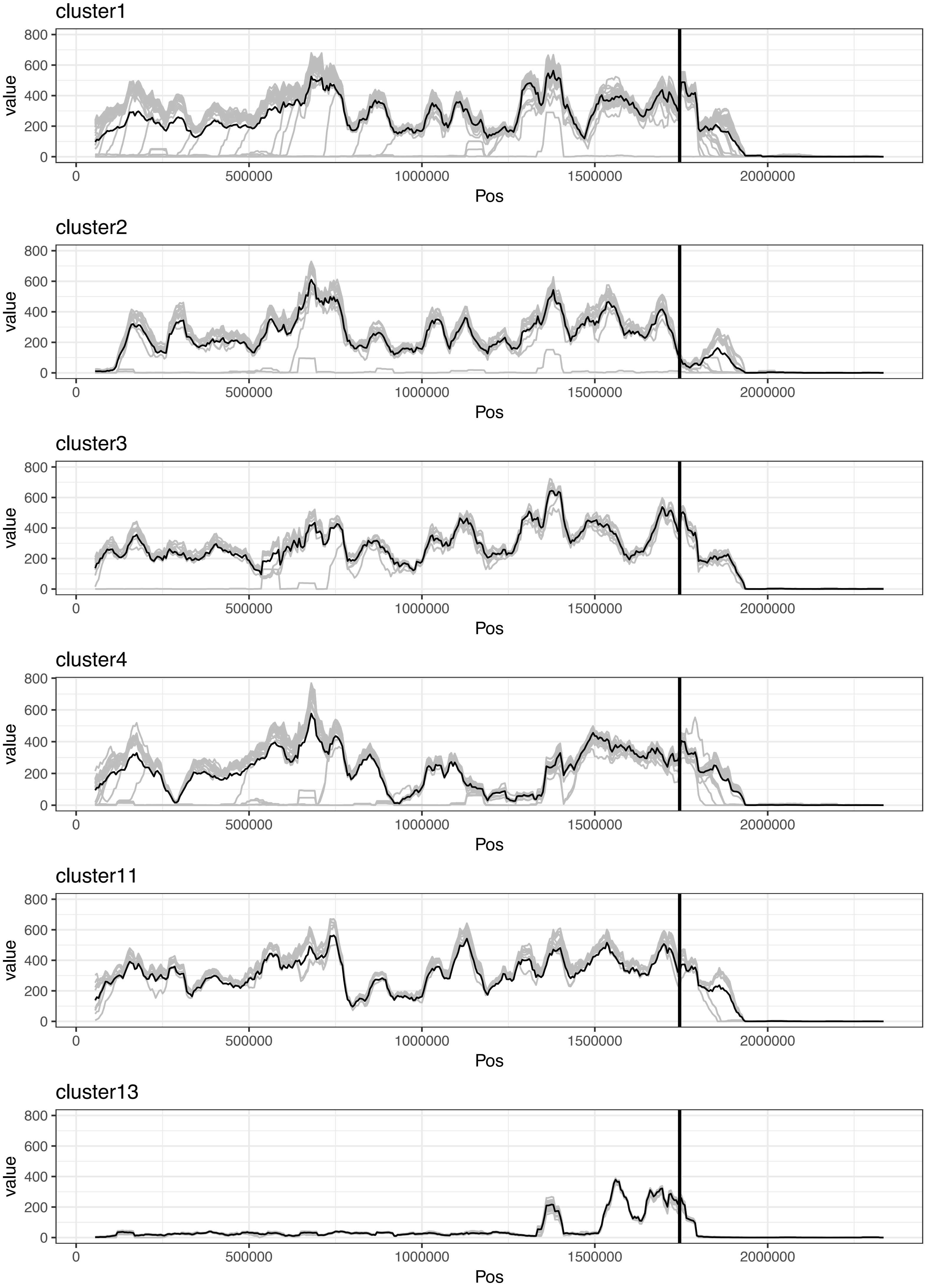

Supplementary Fig. 7: Heterozygosity plots by chromosome and by cluster (for the six most represented clusters 1, 2, 3, 4, 11 and 13. Counts of heterozygous SNPs per windows of 50,000bp were calculated for each strain with a sliding window step of 5,000bp. Plots were generated using the R package ggplot2.

Fst	Cluster 2	Cluster 3	Cluster 4	Cluster 11	Cluster 13
Cluster 1	0,8	0,81	0,83	0,81	0,87
Cluster 2	X	0,77	0,81	0,78	0,87
Cluster 3	X	X	0,82	0,79	0,89
Cluster 4	X	X	X	0,83	0,89
Cluster 11	X	X	X	X	0,9

Supplementary Table 1 (related to Fig. 2): Fixation indices (FST) between clusters calculated using ANGSD.

	pi
Cluster 1	0.478
Cluster 2	0.254
Cluster 3	0.386
Cluster 4	0.256
Cluster 11	0.427
Cluster 13	0.140

Supplementary Table 2 (related to Fig. 1 and 2): Nucleotide diversity (Π) within clusters calculated with samtools.

Polymorphism	Cluster 1	Cluster 2	Cluster 3	Cluster 4	Cluster 11	Cluster 13
**Cluster 1**	28632	4989	4696	4765	4438	530
**Cluster 2**	4989	36720	6393	6296	5233	884
**Cluster 3**	4696	6393	27939	5677	4854	697
**Cluster 4**	4765	6296	5677	34028	5081	838
**Cluster 11**	4438	5233	4854	5081	24350	749
**Cluster 13**	530	884	697	838	749	12310

Supplementary Table 3 (related to Supplementary Data 3): Number of polymorphic positions per cluster.

Media	Nutrients	Concentration (g/L)	Weight for 200mL	Volume (L)	Initial concentration
**VSM (Vaginal Simulative Medium)**	NaCl			0,0116	5mM
	KOH			0,036	2,8g->100mL=0,5M
	Glycerol			0,000128	99%
	Ca(OH)2			0,2	0,15g->200mL=0,01M
	Urea			0,0134	3g->100mL=0,5M
	Glucose			0,066	9g->100mL=0,5M
	YNB			weight: 6,7g	solid YNB
	Acetic acid			0,0085	2M
	Lactic acid			0,00192	Pure
**SSM (Saliva Simulative Medium)**	KCl	0,72	0,144		
	CaCl2(H2O)2	0,22	0,044		
	NaCl	0,6	0,12		
	KH2PO4	0,68	0,136		
	Na2HPO4	0,343	0,0686		
	KHCO3	1,5	0,3		
	KSCN	0,06	0,012		
	Citric acid	0,03	0,006		
	Agar	20	4		

Supplementary Table 4: Composition of vaginal simulative medium (VSM) and saliva simulative medium (SSM).

ORFs	Primer pairs	Side
AFG1	GCCGCGTGTTAATCAGAGATC	Forward
	TATAGTGCCACCGCATCTTG	Reverse
BMT6	CACCATACGCTCTACAAAACTCA	Forward
	ATGGAATTGAGCGAGACGTT	Reverse
SFL1	TGTTCTGCAACGGGATTAGG	Forward
	AACCTCAACGTTGCTCCTCA	Reverse
VTA1	AGGAGCACCGAAATATTTACCAG	Forward
	CAAATTTAGCATGCTTTTGCAC	Reverse
ZCF29	CACGGATCTGTTCCTTCACA	Forward
	CGATTCCCAGTAGCATGACATA	Reverse

Supplementary Table 5: Primer pairs used to check the presence of premature stop codons in six ORFs (*AFG1, BMT6, SFL1, VTA1* and *ZCF29*).
