# Supplementary material for: Gene flow contributes to diversification of the major fungal pathogen Candida albicans
Source: Nat Commun. 2018 Jun 8;9:2253. doi: 10.1038/s41467-018-04787-4 (PMC5993739; doi:10.1038/s41467-018-04787-4)
Supplement: Supplementary file 3 — Description of Additional Supplementary Files [file 41467_2018_4787_MOESM3_ESM.pdf]

Description of Additional Supplementary Files

File Name: Supplementary Data 1
Description: **(related to Fig. 1, 2 and 3)** *Candida albicans* genome sequencing summary: information of strain origin, sequencing technology, cluster assignation based on MLST data and sequencing data, MTLa/alpha genotypes, ploidy inferred from DNA content analysis, mean coverage, number of mapped reads, number of heterozygous and homozygous SNPs and indels, segmental chromosomal deletions or amplifications.

File Name: Supplementary Data 2
Description: **(related to Fig. 1)** SNP dataset encompassing 589,255 SNPs where some of the filters described in the Method could be not respected. A code was created to have information of which filter did not pass: -- for wrong allelic ratio of reference/alternative allele for heterozygous positions, ++ for wrong allelic ratio of reference/alternative allele for homozygous positions, ## for a read depth not comprised between 0.5 and 1.5 of the mean genome coverage; some positions could have several filters which did not pass: a combination of -- and ## gave && and ++ and ## gave **.

File Name: Supplementary Data 3
Description: **(related to Fig. 1, 2, 3, 4)** SNP dataset encompassing 264,999 highly-confident SNPs across the 182 isolates containing no missing data.

File Name: Supplementary Data 4
Description: **(related to Fig. 3)** PhiTests results calculated by ORFs calculated with PhiPack.

File Name: Supplementary Data 5
Description: Analyses of variance (ANOVA) and post-ANOVA comparisons (Tukey's HSD tests) on morphology size scores generated by Iris for colony growth on different media (YPD, VSM and saliva) at different temperatures (18°C, 30°C, 37°C and 42°C).

File Name: Supplementary Data 6
Description: Premature stop codons by ORFs in the 6 most represented clusters, i.e. clusters 1, 2, 3, 4, 11 and 13. First sheet: counts of premature stop codons in the 221 ORFs having a premature stop codon in at least one strain; Second sheet: Number of shared premature stop codons by x strains; Third sheet: Features of the 39 ORFs with premature stop codons specific to cluster 13.

File Name: Supplementary Data 7
Description: Morphology size scores generated by Iris for colony growth for the 182 *C. albicans* isolates on vaginal simulative medium (VSM) and YPD at different temperatures (18°C, 30°C, 37°C and 42°C). For saliva simulative medium (SSM), Iris could not detect limits of colonies and we measured diameters of colonies (mean of two perpendicular measures per colony) to obtain a size of colonies.
